# Pregnane X receptors regulate CYP2C8 and P‐glycoprotein to impact on the resistance of NSCLC cells to Taxol

**DOI:** 10.1002/cam4.960

**Published:** 2016-11-22

**Authors:** Yan Chen, Wandan Huang, Feiyu Chen, Guoping Hu, Fenglei Li, Jianhua Li, Aiguo Xuan

**Affiliations:** ^1^ Department of RespiratoryLiwan Hospital of Guangzhou Medical UniversityGuangzhouGuangdong510170China; ^2^ Department of AnatomyGuangzhou Medical UniversityGuangzhouGuangdong511436China; ^3^ Department of RespiratoryThe Third Affiliated Hospital of Guangzhou Medical UniversityGuangzhouGuangdong510170China; ^4^ Department of PhysiologyGuangzhou Medical UniversityGuangzhouGuangdong511436China; ^5^ Key Laboratory of Neurogenetics and Channelopathies of Guangdong Province and the Ministry of Education of ChinaCollaborative Innovation Center for Neurogenetics and ChannelopathiesGuangzhouGuangdong510260China

**Keywords:** CYP2C8, NSCLC, P‐gp, PXR, resistance, Taxol

## Abstract

Cytochrome P450 2C8 (CYP2C8) is one of the enzymes that primarily participate in producing metabolisms of medications and P‐glycoprotein (P‐gp) has been regarded as one of the important molecules in chemotherapeutically induced multidrug resistance (MDR). In addition, the pregnane X receptor (PXR) is involved in regulating both CYP2C8 and P‐gp. We aim to research the effect of PXR on Taxol‐resistant non–small‐cell lung cancer (NSCLC cells) via regulating CYP2C8 and P‐gp. NSCLC cells were treated with SR12813, LY335979, or PXR siRNA. Cell counting kit (CCK‐8) assay was used to detect cell vitality. Colony formation assay was used to observe cell proliferation. Western blotting, real‐time polymerase chain reaction (RT‐PCR), and immunofluorescence staining were conducted to analyze the expressions of PXR, CYP2C8, and P‐gp. Taxol and its metabolic products were detected by high‐performance liquid chromatography (HPLC). The expression of PXR in A549 cell line was higher than that in other cell lines. The accumulation of PXR was observed in the nucleus after cells were treated with SR12813. Besides, SR12813 induced higher expressions of CYP2C8 and P‐gp proteins. We also discovered that pretreatment with SR12813 reversed the inhibition of cell viability and proliferation after the Taxol treatment in comparison to the SR12813 untreated group. Furthermore, the hydroxylation products of Taxol analyzed by HPLC were increased in comparison to the SR12813 untreated group, indicating that high expressions of CYP2C8 and P‐gp enhanced the resistance of A549 cells to Taxol. For cells treated with PXR siRNA, cell viability, cell proliferation, and Taxol metabolites were significantly reduced after the Taxol treatment in comparison to the siRNA‐negative group. The cell viability, cell proliferation, and Taxol metabolites were regulated by the expressions of PXR, P‐gp, and CYP2C8. That is, PXR expression has an important effect on the resistance of NSCLC cells to Taxol via upregulating P‐gp and CYP2C8.

## Introduction

Lung cancer is a major global health problem and the leading cause of cancer‐related death. One of the main causes of lung cancer is smoking, which is the causal factor in 90% of patients 1. Non–small‐cell lung cancer (NSCLC) constitutes approximately 85% of lung cancer cases and it is the leading malignant cause of cancer‐related mortality all over the world 2, 3. The factors that influence lung cancer therapies include the type of cancer, the time of cancer progression, and the physical status of patients 4. Current treatments involve nonspecific, conventional chemo‐ and radiotherapy regimens and their combination, which results in only a modest increase in survival 5. Despite significant progress has been made in treatment, the therapeutic outcome is not satisfactory and the relapse rates of NSCLC still remain high due to resistance to these interventions 6. Therefore, novel approaches are urgently needed to overcome the resistance in NSCLC patients.

Among all chemotherapeutic agents, paclitaxel (Taxol) has shown great efficacy against various cancer types 7, 8, 9. Taxol is a tubulin‐disrupting agent, which is derived from the bark of the Pacific Yew tree, and it is one of the most commonly used ingredients in anticancer agents. It has shown great efficacy in managing cancers such as ovarian cancer, breast cancer, lung cancer, and other solid tumors such as NSCLC 10, 11, 12, 13. Several potential adverse effects may occur after patients are treated by Taxol initially, including anaphylactic reactions, oropharyngeal mucositis, leukopenia, and resistance to medications 14. The occurrence of resistance to Taxol will be a barrier to successful therapy 15. Additionally, the mechanism that causes drug resistance has not been fully elucidated.

Pregnane X receptor (PXR), which is a member of the nuclear hormone receptor family, has functions including steroid/hormone mediation, xenobiotic and cholesterol metabolism and regulation, and tumorigenesis 16, 17. Previous research has suggested that PXR activation could reduce drug sensitivity and enhance chemoresistance in human cancers through drug‐metabolizing enzymes such as cytochrome P450 2C8 (CYP2C8) as well as ATP‐dependent drug efflux pumps such as P‐glycoprotein (P‐gp) 18. CYP2C8, which is a phase I enzyme, has been shown to have a significant role in the metabolization of Taxol 19. Besides, as an ATP‐dependent drug efflux pump, P‐gp is known to transport and efflux molecules such as Taxol, to limit the intracellular concentration and reduce the biological activity of drugs, resulting in drug resistance as a consequence of its overexpression 20. Studies have shown that CYP2C8 and P‐gp were regulated by activated PXR 21, 22, 23. But up to now, few studies have focused on the relationship among PXR, CYP2C8, P‐gp, and their effects on the resistance of NSCLC patients to Taxol.

In this study, we determined whether PXR plays a role in the resistance of NSCLC cells to Taxol. Since P‐gp and CYP2C8 are important PXR target genes, we aimed to research the effect of PXR on the resistance of NSCLC cells to Taxol via regulating CYP2C8 and P‐gp.

## Materials and Methods

### Cell culture

The normal human lung epithelial cell line HEAS‐2B and the NSCLC cell lines A549, NCI‐H358, HCC827, NCI‐H1650, and NCI‐H1299 were all obtained from BeiNaChuangLian Biological Technology Co., Ltd. (Beijing, China). Cells were cultured in Dulbecco's modified Eagle's medium (DMEM, Corning) supplemented with 10% fetal bovine serum (Gibco) at 37°C with 5% CO_2_ under humidified atmosphere.

### Cell transfection

A549 cells were seeded in six‐well plates at 1 × 10^5^/mL. After incubation for 24 h, cells were transfected with 50 nmol/L PXR siRNA (purchased from RiboBio Co., Ltd., Guangzhou) using Lipofectamine^™^ 2000 Transfection Reagent (Invitrogen) according to the manufacturer's instructions.

### Quantitative real‐time PCR

Total RNA extractions from cells were conducted by Trizol reagent kit (Ambion) under the manufacturer's descriptions. Complementary DNA (cDNA) was acquired using the reverse transcription kit (Fermentas). Real‐time quantitative PCR (RT‐PCR) assay was conducted by quantitative PCR instrument (Fermentas), and the primers are shown in Table 1. The reaction conditions were as follows: predegeneration for 10 min at 95°C, degeneration for 10 sec at 95°C, annealing for 20 sec at 60°C, and extension for 35 sec at 72°C. The relative expression quantity was calculated using the 2^−△△Ct^ method. Relative mRNA expression was estimated by normalization with GAPDH expression. The experiment was repeated for three times.

**Table 1:** Primer sequences of PXR, GAPDH, P‐gp, and CYP2C8 for implementation of RT‐PCR

Gene		Primer sequence
*PXR*	Sense	5′‐ACCTTTGACACTACCTTCTCCCAT‐3′
	Antisense	5′‐CGCAGCCACTGCTAAGCA‐3′
*GAPDH*	SenseAntisense	5′‐GAAGGTGAAGGTCGGAGTC‐3′5′‐GAAGATGGTGATGGGATTTC‐3′
*P‐gp*	Sense	5′‐TGCTCAGACAGGATGTGAGTTG‐3′
	Antisense	5′‐TAGCCCCTTTAACTTGAGCAG‐3′
*CYP2C8*	Sense	5′‐CACCATGGAACCTTTTGTGGTCC‐3′
	Antisense	5′‐TCAGACAGGGATGAAGCAGA‐3′

PXR, pregnane X receptor; GAPDH, glyceraldehyde phosphate dehydrogenase; RT‐PCR, real‐time polymerase chain reaction.

### Western blot

Cells were collected and washed with cold phosphate‐buffered saline (PBS) twice, and resuspended in RIPA Cell Lysis Buffer (Gefan Biotechnology, Shanghai, China) for at least 30 min on ice. The supernatant of lysate was collected after centrifuging at 13,000*g* for 5 min at 4°C. The protein concentration was determined by the bicinchoninic acid (BCA) method. Equal amounts of protein were separated with sodium dodecyl sulfate polyacrylamide gel electrophoresis (SDS‐PAGE) gels and subsequently transferred to polyvinylidene fluoride (PVDF) membranes. Membranes were blocked with 5% nonfat milk for 1 h at room temperature, and then incubated overnight at 4°C with primary antibodies for PXR and GAPDH (1: 300, 1: 800 dilution, respectively), GAPDH as a normalizer. Membranes were incubated with peroxidase‐conjugated secondary antibodies (1: 900 dilution) for 2 h at room temperature and then the signals were developed using an enhanced chemiluminescent detection kit. All antibodies were purchased from Abcam Biotechnology Company.

### CCK‐8 assay

Cell viability was detected by the cell counting kit 8 (CCK‐8) assay according to the manufacturer's protocol (Dojindo, Japan). Cells were seeded in 96‐well plates at a density of 4 × 10^3^ cells/mL and incubated overnight for attachment. After treated with drugs for indicated concentrations and times, cells were incubated with CCK‐8 solution for 3 h at 37°C. The optical density (OD) value was measured at an absorbance wavelength of 450 nm.

### Colony formation assay

The colony formation assay was used to detect the fractions of living cells after exposure to medications. Briefly, A549 cells were seeded in 12‐well plates at a density of 1 × 10^3^ cells per well. Cells were incubated with Taxol for 24 h after being pretreated with SR12813 for 12 h. Thereafter, cells were cultured with fresh DMEM solution for 7 days and the Giemsa‐stained colonies were enumerated.

### High‐performance liquid chromatography

The collected cells were centrifuged and the supernatant was extracted by ethyl acetate. The freeze drying powders were then dissolved in 200 *μ*L methyl alcohol/water solutions (65:35, v/v). Taxol and 6′‐hydroxyl Taxol were analyzed by high‐performance liquid chromatography (HPLC) assay (injection volume: 20 *μ*L, analytical column: C18, eluent: methyl alcohol/water (65:35, v/v), flow rate: 1 mL/min).

### Immunofluorescent staining

Cells were seeded in 24‐well culture plates at a density of 2 × 10^3^ cells/well and then treated by medications for 48 h. A laser scanning confocal microscope was used to analyze cells immediately after incubation with nucleus dye (DAPI), and antibodies for PXR, CYP2C8, and P‐gp, in accordance with the manuscript of immunofluorescent staining detection kit (Leagene Biotechnology Co., Ltd, Beijing).

### Statistical analysis

GraphPad Prism 6.0 software (GraphPad Software, La Jolla, CA, USA) was used to perform statistical analysis. All experiment results were expressed as means ± and standard deviations (SD). Multiple sets of data were compared using the one‐way ANOVA or Student's *t*‐test (two‐tailed). *P *<* *0.05 was considered to be statistically significant.

## Results

### PXR upregulates in NSCLC cell lines

**Figure 1:**
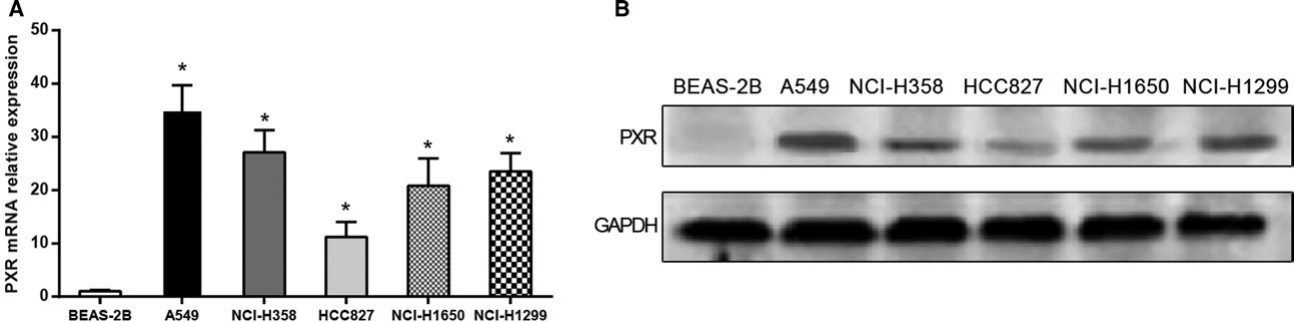
The expression of pregnane X receptor (PXR) in BEAS‐2B cells and five non–small‐cell lung cancer (NSCLC) cell lines. (A) The relative mRNA expression of PXR in different cell lines by RT‐qPCR assay, GAPDH as internal control. (B) The protein expression of PXR in different cell lines analyzed by western blot assay, GAPDH as internal control. Data were presented as mean ± SD for three independent experiments. *P <* *0.05 versus BEAS‐2B cells.

We analyzed the mRNA and protein expressions of PXR in BEAS‐2B, A549, NCI‐H358, HCC827, NCI‐H1650, and NCI‐H1299 cell lines by RT‐PCR and western blot assays. As shown in Figure 1, the mRNA and protein expressions of PXR in NSCLC cell lines especially in A549 cell line were higher than the BEAS‐2B cell line (*P *<* *0.05). Therefore, A549 cells were used in the subsequent experiment.

### Effect of PXR upregulation on the expressions of CYP2C8 and P‐gp

**Figure 2:**
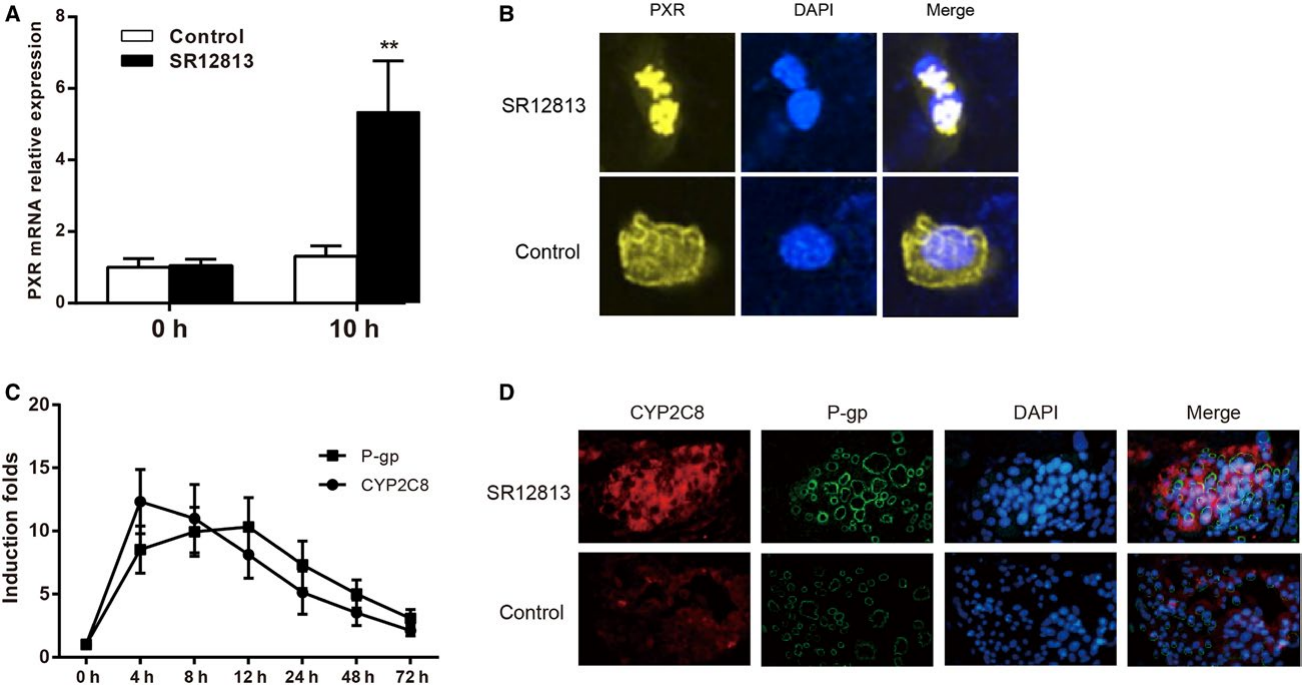
The expressions of CYP2C8 and P‐gp were upregulated after exposure to SR12813 in A549 cells. (A) The relative mRNA expression of pregnane X receptor (PXR) in A549 cells before and after treatment with SR12813, GAPDH as internal control. (B) Quantitation of PXR protein expression using immunofluorescent staining assay in A549 cells with SR12813 treatment, PXR was stained with yellow dye and cell nucleus was stained with blue dye. (C) The levels of mRNA expression of CYP2C8 and P‐gp after SR12813 treatment at different times. (D) Quantitation of CYP2C8 and P‐gp protein expression using immunofluorescent assay in A549 cells with SR12813 treatment, CYP2C8 was stained with red dye, P‐gp was stained with green dye, and cell nucleus was stained with blue dye. Data were presented as mean ± SD for three independent experiments. **P <* *0.01 versus control group.

RT‐PCR and immunofluorescent staining assays were used to analyze the expression of PXR after cells were exposed to SR12813 for 10 h. As shown in Figure 2A and B, cells treated with 1 *μ*mol/L SR12813 for 10 h displayed higher levels of PXR than the untreated groups. Immunofluorescent staining revealed the accumulation of PXR in the nuclei of cells.

In order to observe the expressions of CYP2C8 and P‐gp after cells were treated with 1 *μ*mol/L SR12813, RT‐qPCR was used to detect the variation in mRNA of CYP2C8 and P‐gp at different times. As shown in Figure 2C, the expressions of CYP2C8 and P‐gp were increased after exposure to SR12813. The expression of CYP2C8 was peaked at 4 h after treatment with SR12813, and P‐gp was peaked at 12 h after treatment with SR12813. The results of immunofluorescent staining revealed that the expressions of CYP2C8 and P‐gp were upregulated at 8 h after treatment with SR12813 compared to control group (Fig. 2D).

### PXR enhanced Taxol resistance via upregulating CYP2C8 and P‐gp in A549 cells

**Figure 3:**
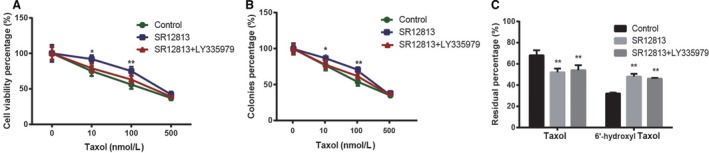
PXR enhanced Taxol resistance in A549 cells. (A) The viability of cells was tested by CCK‐8 assay. (B) The proliferation of cells was tested by colony formation assay. (C) The Taxol and 6′‐ hydroxyl Taxol were analyzed by HPLC assay. Data were presented as mean ± SD for three independent experiments. *P <* *0.05, **P <* *0.01 versus control group and SR12813 + LY335979 group. HPLC, high‐performance liquid chromatography. PXR, pregnane X receptor.

Cells were pretreated with 1 *μ*mol/L SR12813 for 8 h and then incubated with different concentrations of Taxol for 24 h. The cell viability was analyzed by CCK‐8 assay. As shown in Figure 3A, for SR12813 pretreated cells, cells treated with lower concentrations (<100 nmol/L) of Taxol displayed higher viability than the control group (*P *<* *0.05). However, this effect on drug resistance disappeared under high dosages (>500 nmol/L) of Taxol treatment. When cells were simultaneously treated with SR12813 and P‐gp inhibitor LY335979, cell viability reduced noticeably compared to those treated with SR12813 alone (*P *<* *0.05). The colony formation assay also demonstrated that after pretreated with SR12813, A549 cells had higher colony formation efficiency than the control group under lower concentrations (<100 nmol/L) of Taxol treatment, but this drug resistance ability was disappeared under high dosages (>500 nmol/L) of Taxol treatment. The colony formation efficiency reduced noticeably after treated with SR12813 and P‐gp inhibitor LY335979 compared to those treated with SR12813 alone (*P* < 0.05) (Fig. 3B). These data demonstrated that upregulated PXR expression increased the resistance of A549 cells to lower concentrations (<100 nmol/L) of Taxol via upregulated P‐gp in A549 cells. In addition, HPLC was used to detect levels of Taxol and its metabolic product “6′‐hydroxyl Taxol” in A549 cells that were pretreated with 100 nmol/L Taxol for 24 h. As shown in Figure 3C, the content of Taxol in 1 *μ*mol/L SR12813 treated groups was remarkably lower than untreated groups (*P *<* *0.05). Besides, the content of 6′‐hydroxyl Taxol was remarkably higher in SR12813 treated groups than untreated groups (*P *<* *0.05). However, the combined treatment of LY335979 and SR12813 had less effect on the metabolism of Taxol compared to SR12813 treatment alone (*P *>* *0.05). Those results demonstrated that upregulated PXR expression increased the metabolism of Taxol via upregulated CYP2C8 in A549 cells.

### PXR decreased Taxol resistance via downregulating CYP2C8 and P‐gp in A549 cells

**Figure 4:**
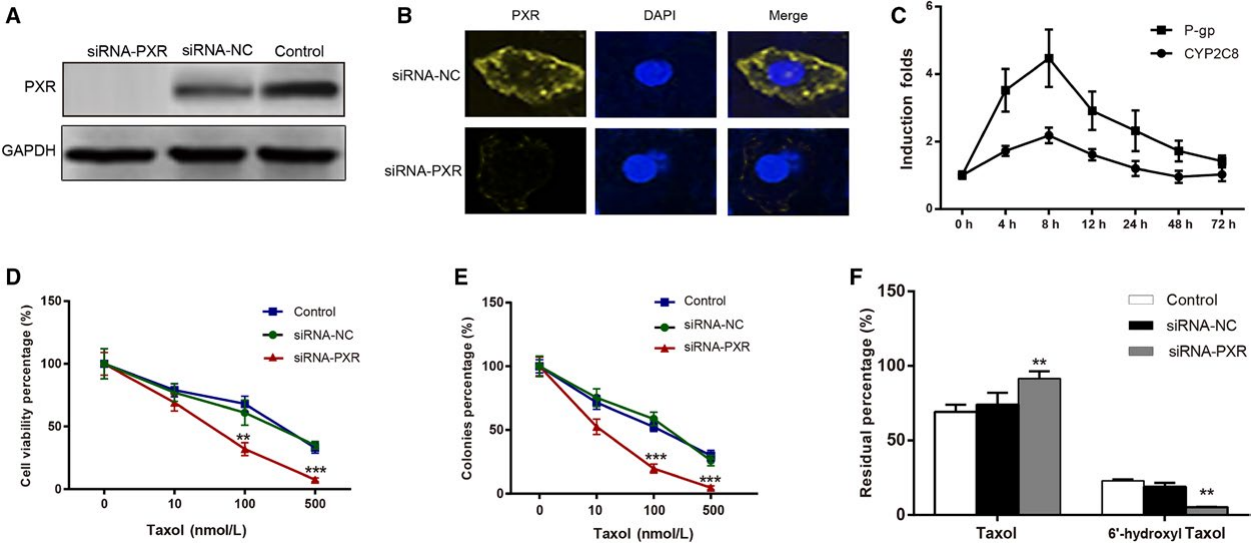
Downregulation of pregnane X receptor (PXR) enhanced the sensitivity of A549 cells to Taxol. (A) Western blotting result confirmed that the PXR expression was significantly lower as compared with untreated and control group after PXR siRNA transfection in A549. (B) Quantitation of PXR protein expression using immunofluorescent assay in A549 cells after PXR siRNA transfection, PXR was stained with yellow dye and nucleus was stained with blue dye. (C) The mRNA expression levels of CYP2C8 and P‐gp in cells with treatment of SR12813 and PXR siRNA. (D) The viability of cells was tested by CCK‐8 assay. (E) The proliferation of cells was tested by colony formation assay. (F) The Taxol and 6′‐hydroxyl Taxol were analyzed by HPLC assay. Data were presented as mean ± SD for three independent experiments. **P <* *0.01, ***P <* *0.001 versus siRNA‐NC group. HPLC, high‐performance liquid chromatography.

We next investigated the effect of PXR knockdown on the expressions of CYP2C8 and P‐gp. As shown in Figure 4A and B, western blot and immunofluorescent staining displayed no obvious level of PXR protein in the siRNA‐PXR group, suggesting that we had expression downregulation of PXR with high efficiency. After downregulation of PXR, A549 cells were treated with 1 *μ*mol/L SR12813, and the mRNA expression of CYP2C8 and P‐gp was detected by RT‐qPCR. The results showed that the upregulation expression of CYP2C8 and P‐gp by 1 *μ*mol/L SR12813 was downregulated after the decreased expression of PXR by siRNA (Figs. 2C and 4C).

A549 cells were treated with different concentrations of Taxol in siRNA‐PXR, siRNA‐NC, and control groups, and the CCK‐8 assay was used to compare the cell viability of A549 cells. As shown in Figure 4D, there is no significant difference in cell viability between the siRNA‐NC and control groups after treated with different concentrations of Taxol (*P *>* *0.05). However, the cell viability in the siRNA‐PXR group was significantly lower than that of the siRNA‐NC group (*P *<* *0.05). The colony formation assay also demonstrated that the colony formation efficiency in the siRNA‐PXR group was significantly lower than that of the siRNA‐NC group after treated with different concentrations of Taxol (*P *<* *0.05) (Fig. 4E). These data demonstrated that downregulated PXR expression decreased the resistance of A549 cells to Taxol.

Then, we detected the 6′‐ hydroxyl Taxol and Taxol with HPLC assay in siRNA‐PXR, siRNA‐NC, and control groups. As shown in Figure 4F, there is no significant difference in the content of Taxol and 6′‐ hydroxyl Taxol between the siRNA‐NC and control groups (*P *>* *0.05). However, the content of Taxol in the siRNA‐PXR group was significantly higher than that of the siRNA‐NC group (*P *<* *0.05), while the content of 6′‐ hydroxyl Taxol in the siRNA‐PXR group was significantly lower than that of the siRNA‐NC group (*P *<* *0.05). Those results demonstrated that downregulated PXR expression decreased the metabolism of Taxol via downregulated CYP2C8 in A549 cells.

## Discussion

Lung cancer is a malignant disease that is prevalent worldwide. The majority of lung cancers are NSCLC, which accounts for more than 85% of existing cases of lung cancer 24. Even if the primary treatment for NSCLC is surgery or radiation therapy, most patients diagnosed at an advanced stage require chemotherapy which is able to extend the survival status of patients 25, 26, 27, 28. But resistance to most cytotoxic drugs has become a major obstacle in treating the advanced NSCLC 29. As a result of this, researchers have devoted to revealing the corresponding mechanism that may trigger the resistance of NSCLC patients to taxol medications. These studies have listed PXR, cytochromes P450 (CYPs), and P‐gp as the potential targets that are related to drug resistance 30, 31.

In our study, both protein and mRNA expressions of the PXR in human NSCLC A549 cells were significantly higher than the normal lung cells, as well as other NSCLC cell lines. Likewise, in previous studies, compared with normal tissues like bronchial epithelium and alveolar tissues in the normal lung, the neoplastic tissues such as NSCLC cells expressed the PXR at a significantly higher level, which suggested that PXR selectively participated in the development of tumorigenesis 32. Furthermore, PXR participates in resistance to chemotherapeutics by regulating the expression of some enzymes or transports that are associated with the metabolism of the drug 33, 34, 35. Therefore, PXR can be used to predict the potential risk of drug resistance in NSCLC patients.

Recently, several studies inspecting PXR had effect on CYPs and P‐gp. Gahrs et al. have shown that the activation of PXR can govern the expression of CYPs, a member of monooxygenase superfamily, in cultured rat hepatocytes after induced by the cytotoxic drugs 31. Moreover, CYP2C has been proven to be a major enzyme that is used to metabolize Taxol in the previous study 36. Besides, a number of studies have demonstrated that P‐gp, a plasma membrane transporter, leads to multidrug resistance of liver carcinoma. In addition, studies have shown that ABC transporter protein, containing P‐gp proteins involved in multidrug resistance (MDR), are associated with the development of drug resistance via the regulation of PXR in small molecule tyrosine kinase inhibitors 37, 38, 39.

We used SR12813, the agonist of PXR, to enlarge the effect of activation of PXR on A549 cells to verify our hypothesis that PXR enhances the drug resistance. When the A549 cells were treated with SR12813, we were surprised to find the upregulation of the expressions of CYP2C8 and P‐gp as well as the improvement in the drug resistance, including the increased of cell viability, cell proliferation, and cellular ability to metabolize Taxol. Our results consisted with the previous findings that CYPs and P‐gp were induced in human liver cell and colon cell lines by the activation of PXR 40, 41. Similar results were also found in ovarian cancer cell lines by Gupta et al. 42.

After transfecting PXR siRNA into A549 cells to downregulate PXR, we also used SR12813 to stimulate PXR expression. Interestingly, SR12813 still significantly induced the expression of P‐gp, but had less effect on the expression of CYP2C8 compared with that of P‐gp. We speculated that SR12813 enhanced the induction of P‐gp not only by upregulating PXR, but also by modulating other signal pathways. Those findings further validated that PXR is the regulator of CYP2C8 and P‐gp, which are associated with the resistance of NSCLC cells to Taxol. In addition, a recent study by Melguizo et al. also emphasized that the gene expression of MDR1 was associated with PXR in the progression of drug resistance in treating NSCLC cell lines 43. Thus, drug resistance may be attuned in different pathways via the activation of PXR and further studies should be conducted to confirm it.

To sum up, our study demonstrated that the activation of PXR stimulates the resistance of NSCLC cell lines to Taxol by targeting the induction of CYP2C8 and P‐gp. Selectively downregulating the expression of PXR may help clinicians to improve the sensitivity of NSCLC cells and enhance the effects of anticancer medications on NSCLC patients.

## Conflict of Interest

None declared.
